# Filling nanopipettes with apertures smaller than 50 nm: dynamic microdistillation

**DOI:** 10.3762/bjnano.9.204

**Published:** 2018-08-16

**Authors:** Evelyne Salançon, Bernard Tinland

**Affiliations:** 1 Aix Marseille Univ, CNRS, CINAM, Marseille, France

**Keywords:** current rectification, distillation, filling, nanopipette

## Abstract

Using nanopipettes with very small apertures (<10 nm) is a good way to improve the spatial resolution in scanning conductance experiments, to monitor single-molecule delivery and to strain long molecules stretching during translocation. However, such nanopipettes can be difficult to fill. Here we describe a dynamic microdistillation technique that successfully fills all nanopipettes, whatever their shape or tip radius. Even elongated or bent nanopipettes with a small-angle tip are completely filled using this new technique. The nanopipettes are first filled with pure water, which is later replaced with the desired electrolyte via electromigration. Electrical measurements are used to check that filling is complete.

## Introduction

Nanopipettes and nanocapillaries are often used as a manipulation tool for single molecules in liquids. A wide range of studies use capillaries with nanometer-sized apertures [1–6]. For example, simulated translocation with nanopipettes is currently being explored worldwide [7]. A large community of researchers is currently working on characterizing nanopipette geometries, using different methods to address aperture size [8–13]. While nanopipettes are known to be simple and easy to fabricate, batch-to-batch reproducibility is poor, particularly for the difficult-to-obtain small-hole nanopipettes. Electrical characterizations depend on the complete filling of nanopipettes, which means no gas bubbles inside, to ensure good conductivity. The capacity to fill all types of nanopipettes, including small-hole nanopipettes (<10 nm), bent nanopipettes, with both small and large angles, would therefore be highly desirable. It would help to improve the spatial resolution in scanning conductance experiments, in optimizing the signal-to-noise ratio during single-molecule delivery and in straining long-molecule stretching during translocation.

Small nanopipette apertures make backfilling unfeasible. There are many other ways to fill nanopipettes, but none is very efficient. One way is to apply high pressure behind the fluid placed in the large part of the capillary. Conversely, the so-called desiccator method consists in applying a depression of about 1mbar in the external environment. A third solution is to use a nanopipette with a glass filament inside. The filament is melted during tape manufacturing, which can lead to glass impurities in the capillary and geometrical irregularities at the very end of the nanopipette. In some cases, the intended use of the nanopipette, especially the translocation of objects, can introduce undesired microfluidic inhomogeneities, even contributing to aperture clogging. The fourth method, plasma treatment, involves improving the wettability by hydrophilizing the glass surface in an O_2_ plasma [8–9]. Zweifel et al. [8] observed that electrical (*I*–*V*) curves were strongly modified, possibly due to surface chemistry modifications such as reactive hydrogen activation. Regardless of whether this is intentional or not, and considered an advantage or a drawback, the initial surface chemistry seems to be changed by the treatment and probably also the surface roughness. Moreover, the very small aperture range, our particular focus, was not explored in this work.

When high back-pressure is applied to the filling fluid, we showed in a previous article that the fluid meniscus follows an air-flow Poiseuille law [14] enabling the size of the hole to be determined. However, given the filling velocities, filling capillaries this way is very time-consuming. Our previous study found that for elongated nanopipettes with a tip longer than 10 mm and high back-pressure of about 2.5 bar applied, the meniscus velocity is about 0.02 μm/s for a nanopipette with a diameter of 40 nm. Filling can thus take several days (about six days in the present example), even though the velocity increases with decreasing diameter. In a recent article, S. Hennig [15] even wrote that “it can be very difficult, if not impossible, to fill a nanopipette with a small diameter of 10 to 20 nm and an extended taper length of 4 to 5 mm, even if glass capillaries with filament are used”.

Here, we first show that traditional methods are ineffective in filling certain kinds of nanopipettes, especially elongated and small-hole nanopipettes. Second, we demonstrate that the new filling method, dynamic microdistillation, is capable of filling any capillary. Filling time, complete filling and electrical equilibrium were assessed on “basic nanopipettes”, without modification of their internal geometry or chemistry. The nanopipettes used have an inner diameter as low as 7 nm, without filaments. They were filled, without plasma treatment and using a simple method. This nanopipette filling process is usable for all types of nanopipettes. Details of some are provided in this paper, together with the method and reproducible electrical measurements to prove complete filling.

## Results and Discussion

Quartz capillaries, being of pure silica, have certain intrinsic properties (low dielectric constant, low loss factor, high volume resistivity, strength and chemical purity) that make them appropriate for the reproducible electrical measurements described below. Notably, they eliminate ion leakage, and offer the lowest possible background noise levels in electrical current recording. The capillaries, the puller and the set of parameters used to obtain nanopipettes are described below in the Experimental section.

The general shape of nanopipettes can vary, but tip length and hole diameter are usually in the expected range for a given set of parameters. Due to variations in the builder calibration, this set of parameters can change from one puller to another. The very end of each tip is always different and has to be characterized. Here, we determined tip shape and aperture via different methods: first, by using the velocity of the water meniscus and an optical microscopy view of the tip [14]; second, by using scanning electron microscopy (SEM) after capillary use (this method is destructive); third, via electrical characterization of the nanopipette.

**Figure 1:**
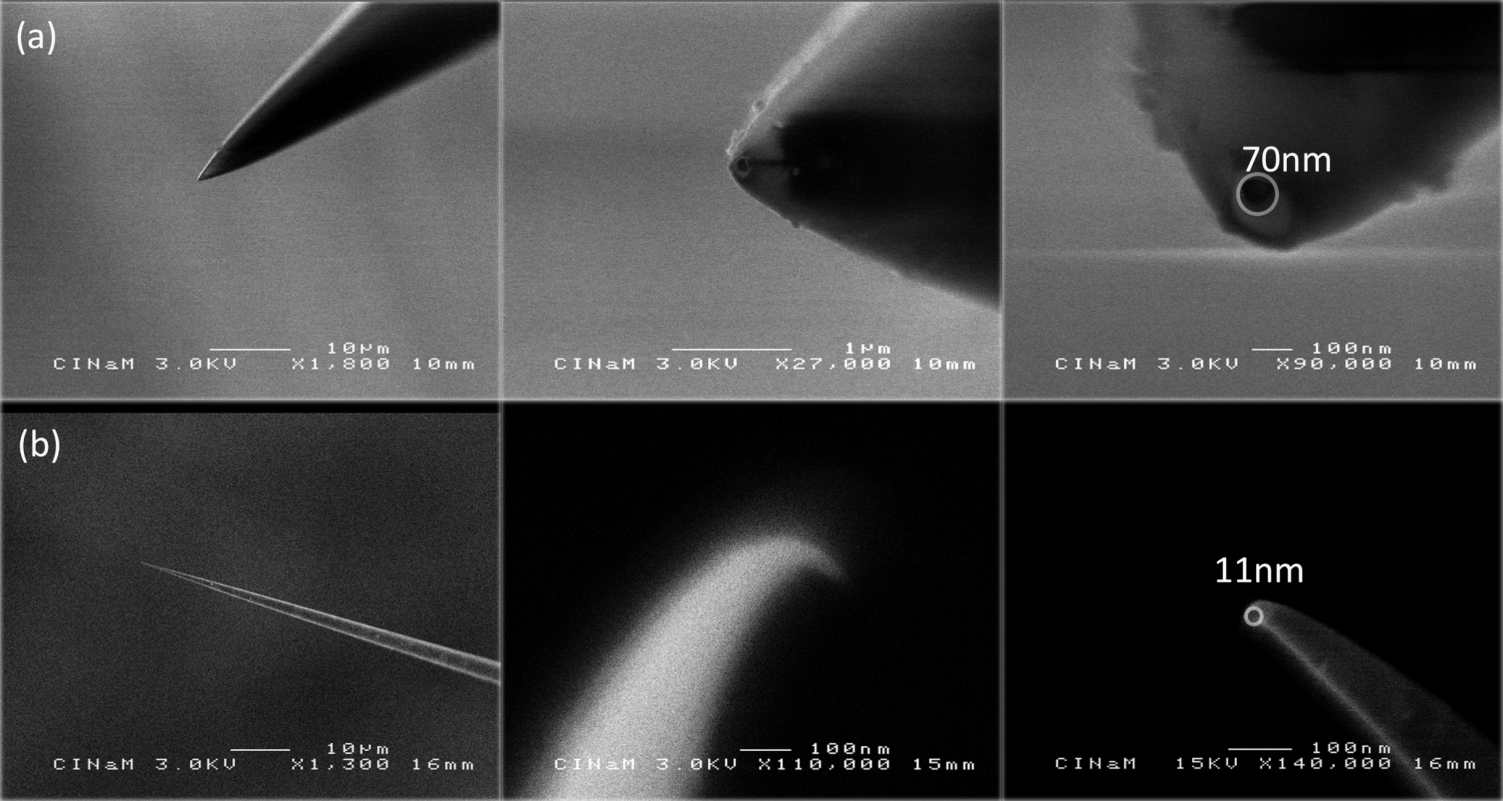
Upper line (a): three SEM images of the same nanopipette filled by applying high back-pressure; lower line (b): three SEM views of a capillary impossible to fill by applying high back-pressure (see fabrication program 2, Experimental section). It is very thin, elongated and bent at its tip with a hole of *d*_tip_ = 11 nm.

Figure 1 shows the SEM view of two typical nanopipettes. The nanopipette in Figure 1a was filled by applying high back-pressure. The nanopipette in Figure 1b, which was impossible to fill by the usual methods, was filled using the technique proposed here. Probably due to a parameter set that favors glass sinking flow before pulling, it is bent at its tip, with an 11 nm hole. This can be viewed as an artefact, but it illustrates the strength of the method. Klenerman et al. [16], at least in their schematic setup, found this design useful. The last angle is invisible in optical microscopy. The nanopipettes that proved impossible to fill here by the high back-pressure method showed these types of defects, which are common. Nanopipettes are still open but the tip shape can be highly irregular. Irregular tip shapes obviously make filling these nanopipettes harder.

Our success in using the high back-pressure method to fill this kind of nanopipette, thin and elongated with a small-angle end tip, was random (roughly 20%), with a high probability of breaking the tip. Actually, what we observed was that filling became very difficult when apertures were smaller than 50 nm. Often, the very end of the capillary quickly became plugged by water condensation or air pollution, which completely and irreversibly stopped the filling. For shorter nanopipettes, the high back-pressure method is efficient but still takes time, several hours or even two or three days. The surrounding atmosphere needs to be particularly clean. Our tests applying high back-pressure applied to filament capillaries were not very successful: filling remained impossible with elongated nanopipettes.

Thus, current nanopipette filling methods appear inefficient, time-consuming and wasteful (up to 80% waste). In section Experimental, we propose a new technique that fills 100% of nanopipettes. This dynamic microdistillation process can be applied to fill any nanopipette, whatever its shape or tip hole. The process is as follows: Due to the heating involved, a quartz nanopipette is recommended. A wall of heat is moved along the nanopipette. Deionized water boils inside the nanopipette and re-condenses to the tip in the cold part. The water plug at the tip grows with the motion of the heat wall. Water has been chosen because it is the solvent of potassium chloride, the salt used for the electrical measurements. Only the very end of the tip is filled with pure water, the micrometric part of the capillary being replaced by the saline solution. Although ion diffusion at the very end of the tip only concerns a negligible volume, in such a confined situation it can take a long time, from hours to days, to reach electrical equilibrium by diffusion alone. Nanopipettes are filled faster than by using high back-pressure, in roughly one hour. The technique works with all nanopipettes, filling even closed nanopipettes. Results are tested directly by electrical conductivity to check that filling is complete, that the nanopipette is open and that electrical equilibrium is reached. The electrical measurement setup is described in the Experimental section.

**Figure 2:**
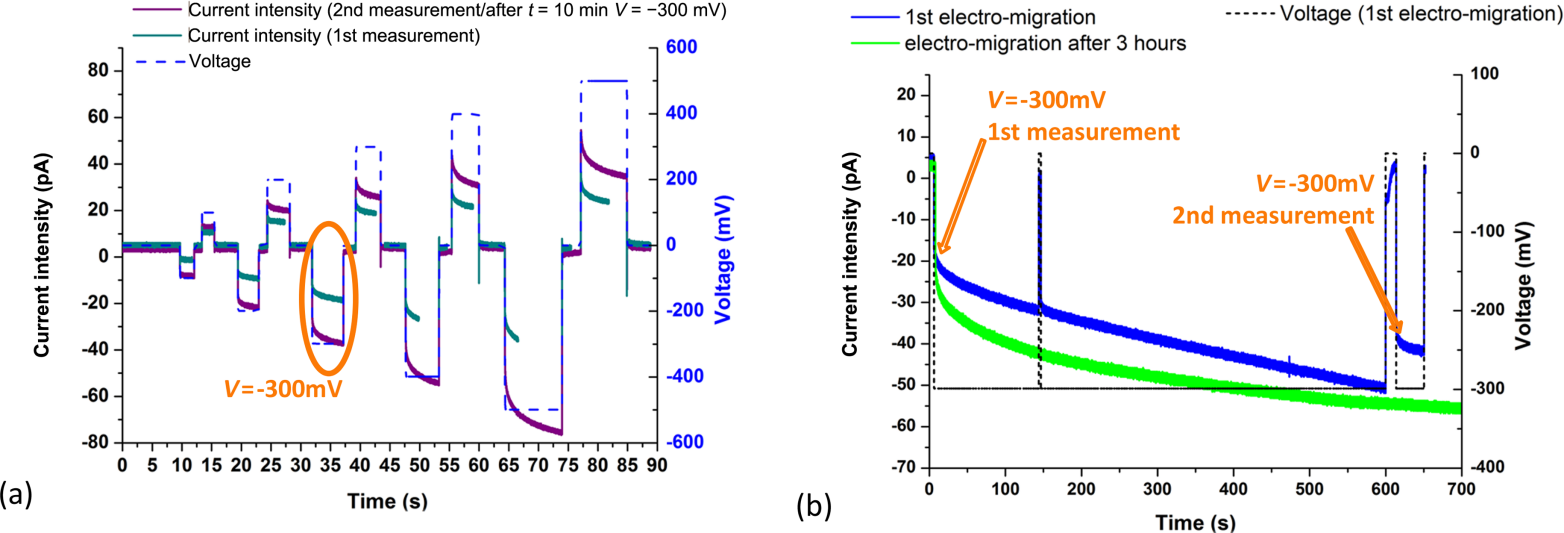
Typical *I*(*V*) measurements with pClamp: *I* and *V* are recorded as a function of the time. [KCl] = 10^−4^ mol·L^−1^ and the geometrical nanopipette characteristics are *r*_tip_ = 13 nm, θ = 9° and *l*_tip_ = 15 mm. (a) The first measurement (cyan) directly after the KCl filling shows the electrical conductivity of the system. The nanopipette is open and filled. The second measurement (purple) after the electromigration shown in (b) shows the nominal current intensity values together with the voltage values. The electrical characteristics are reproducible; (b) Electromigration directly after filling and three hours later, *V* = −300 mV over a period of time of *t* = 10 min.

Figure 2a shows a typical recording made with an elongated, small-angle and small-hole nanopipette: *r*_tip_ = 13 nm, θ = 9° and *l*_tip_ = 15 mm. Voltage supply is increased from 0 to ±500 mV and the current intensity is recorded at the same time. The first measurement is made directly after the first electrolyte loading at a concentration of [KCl] = 10^−4^ mol·L^−1^.

This first electrical measurement confirms that the nanopipette is filled: The current flows and ion exchange is ensured between the Ag/AgCl electrodes. However, the tip of the nanopipette is still filled with pure deionized water. The intensity level does not correspond to the stationary regime, instead it changes slowly with diffusion.

To ensure that electrolyte concentration reaches its nominal value throughout the nanopipette, electromigration is performed (Figure 2b), i.e., ion exchange is enforced through voltage applied for a given time. Here, electromigration is performed by maintaining the power supply at *V* = −300 mV over a period of time of *t* = 10 min. During these 10min, the intensity level increases from *I*_−300mV_ = −30 pA to an upper limit of about *I*_−300mV_ = −55 pA. A second measurement of current intensity as a function of the voltage is then performed (in purple in Figure 2), which yields higher values than the previous one. To check that a stationary regime has been reached, after three hours and a new electromigration process (*V* = −300 mV for 10 min), the current intensity versus voltage measurement is performed again. The obtained values of the electrical current exactly match those of the second measurement (in purple, Figure 2). The repeatability of measurements after several hours confirms that electromigration leads to a homogeneous ion distribution faster and more efficient than diffusion alone.

All nanopipettes filled with this technique were electrically tested. Some “current intensity pairs” as a function of the estimated hole size are given in Table 1. A current intensity pair represents the current intensity values before and after electromigration with an applied voltage of about *V* = −500 mV over a period of time of *t* = 10 min at the given salt concentration. Tip-hole size uncertainties depend on the measurement techniques used and any overlap among measurements.

**Table 1:** Current intensity measurements made at *V* = −500 mV and at the salt given concentration before and after electromigration for *t* = 10 min for different tip-hole sizes.

tip-hole size (nm)	concentration [KCl] (mol·L^−1^)	current intensities *I* (±5 pA) before/after electromigration

109 ± 6	10^−4^	−111/−256
81 ± 5	10^−4^	−56/−135
20 ± 5	10^−2^	−261/−418
15 ± 5	10^−2^	−60/−135
7 ± 1	10^−2^	−34/−75

**Figure 3:**
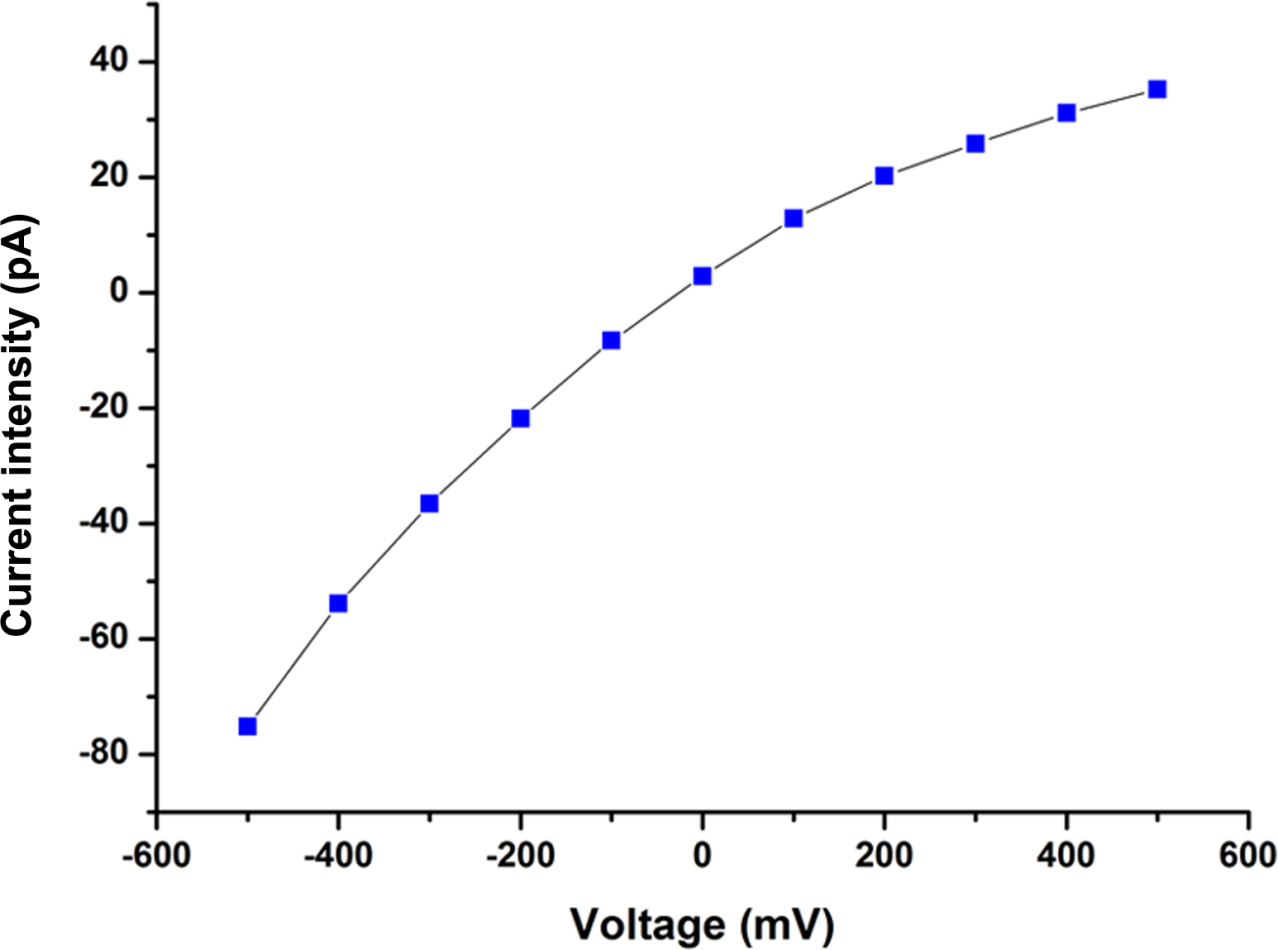
*I*(*V*) at [KCl] = 10^−4^ mol·L^−1^ for a capillary with *r*_tip_ =13 nm, θ = 9° and *l*_tip_ = 15 mm. The current intensity as a function of the voltage was extracted from the result in Figure 2 and exhibits a rectification effect: ICR_500_ = *I*_−500mV_/*I*_+500mV_ = 1.53 at [KCl] = 10^−4^ mol·L^−1^.

All of the more than 20 nanopipettes were filled, regardless of their shape or tip-hole size. All showed a signature related to their shape. In Figure 3, the current intensity versus voltage (*I*–*V*) curve extracted from the data of Figure 2 is given. A rectification effect is observed for this kind of nanopipette [1,10,12–13]. Both current intensity values and ICR (intensity current rectification) ratio of ICR_500_ = *I*_−500mV_/*I*_+500mV_ = 1.53 at [KCl] = 10^−4^ mol·L^−1^ are comparable with previously measured values. This confirms that the proposed filling technique is reliable.

## Conclusion

The successful filling of 100% of nanopipettes using dynamic microdistillation is described here. The process involves growing a plug of deionized water at the tip of the nanopipette by moving the water-filled capillary into the vicinity of a heated filament. The water is brought to a boil inside the capillary and re-condensed. This re-condensed water is deposited on the plug, which grows until it reaches the micrometric part of the capillary.

After complete filling, the water in the large part of the capillary is replaced by the desired electrolyte. Electromigration yields the electrical stationary regime after a few minutes. Here we present an electrical setup used in our experiment, but the stationary regime could be obtained using a more inexpensive power supply.

The nanopipette filling process is usable for all types of nanopipettes, including small-hole nanopipettes (<10 nm), bent nanopipettes, with a small or large angle, and even closed nanopipettes. All filled nanopipettes were open and exhibited electrical characteristics related to their shape. The process works despite surrounding atmospheric pollution and humidity, which usually impede complete filling. Using a programmable piezo-electric actuator, the process could be automated and certainly optimized in terms of a smooth and regular flow.

The overall experimental technique leads to a workable system within a reasonable time. Pulling the nanopipette, determining its size by the Poiseuille technique, filling it and carrying out the electrical measurement takes only a few hours, making the nanopipette reliably usable in any experimental application.

## Experimental

### Nanopipette fabrication

The capillaries used are pure silica tubes (quartz tubes) from Sutter (HERAEUS HSQ300) with 1 mm outer diameter and inner diameters of 300, 500 or 700 µm. Due to their high melting temperature, they are pulled with a P-2000 laser-based micropipette puller from Sutter Instruments. Table 2 shows typical programs used to pull nanopipettes that are likely to produce a small hole (diameter below 100 nm).

**Table 2:** Puller programs with the obtained general shapes and hole diameters *d*_tip_ < 100 nm.

inner diameter of quartz tube (µm) Sutter ref.	heat	filament	velocity	delay	pull	general shape

300 Q100-30-7.5	750	5	65	126	185	*l*_tip_ > 10 mm
700 Q100-70-10	750	5	65	126	185	*l*_tip_ > 10 mm
700 Q100-70-10	700	4	60	145	175	*l*_tip_ < 10 mm
500 Q100-50-10	750	4	53	135	100	*l*_tip_ < 10 mm

### Dynamic microdistillation

**Figure 4:**
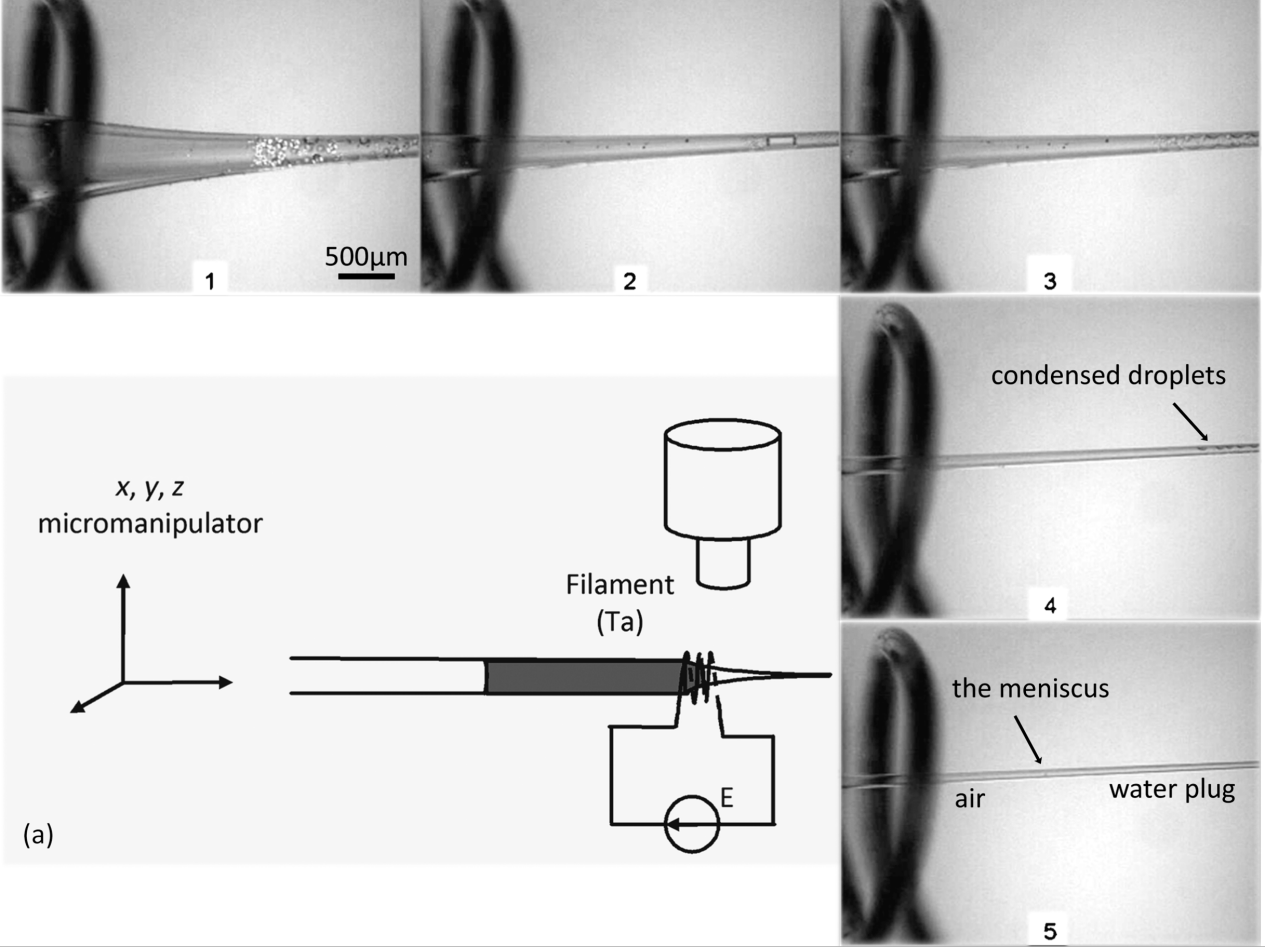
(a) Experimental setup of the new filling technique. The nanopipette is inserted into a tantalum loop (wire diameter: 250 µm, filament diameter and length: 5 mm) powered electrically by a stabilized generator (*V* = 3 V, *I* = 3.5 A). The nanopipette can be moved with a piezoelectric (*x*, *y*, *z*) manipulator and is loaded with water up to its millimeter-to-micrometer region using a micro-syringe. The loop of the filament is placed under an optical microscope. Images 1 to 5 illustrate the process. (1) The heated filament brings the water to a boil inside the capillary; water re-condenses on the cold wall of the nanopipette. (2) The capillary is moved to heat the part where the water has re-condensed as droplets. (3) The droplets are boiled again and water is re-condensed further on the next cold part of the nanopipette. (4) The process is repeated. (5) The water is re-condensed at the very end of the tip; the meniscus formed by the water plug is visible under the optical microscope.

The new technique uses dynamic microdistillation, as shown in Figure 4. The nanopipette is first filled with deionized water up to the accessible micrometer-scaled part of the capillary. A 200 nm syringe filter is used to keep the capillary free of dust. The capillary is held by a (*x*, *y*, *z*) micromanipulator and the water meniscus is positioned in a loop of a tantalum filament under a large-field optical microscope. The filament of 250 µm tantalum wire is 5 mm long and its loop diameter is also about 5 mm. Tantalum was chosen because of its high melting point and its resistance to oxidation. The filament is heated by using the Joule effect at *V* = 3 V, which means at roughly *I* = 3.5 A. The filament becomes white without burning at atmospheric pressure. When the filament is white, the water inside the capillary is brought to a boil. The water vapor re-condenses on the large part of the capillary (left of the tantalum loops), but only slowly because of the heat. The water vapor molecules are not visible since they do not greatly affect the large meniscus. Most of the vaporized water heads for the narrow part of the nanopipette (right side on the pictures) and re-condenses as a string of droplets as soon as it encounters a significantly colder region. Moving the nanopipette inside the loop slowly and regularly away from the meniscus (velocity of about 1 mm/s) either pushes the water vapor with a “moving wall of heat” or re-vaporizes the condensed string of droplets, which re-condenses further. The droplets in the string become smaller and smaller as the tip narrows. The process transports the water up to the very end of the nanopipette by “distillation”. The transported water becomes visible under the microscope because a small meniscus appears and moves gradually toward the large part of the nanopipette (due to the back-and-forth motion of the nanopipette). Paying much attention, the reader can see the thin lines delimiting the droplets and the coming meniscus in Figure 4 (we did not enlarge the line thickness on purpose to maintain a global coherent scale). The process is repeated so that the tip water plug grows until the micrometer-scaled part of the capillary becomes accessible with the micropipette syringe. Then the water in the large part is removed and replaced with the desired electrolyte solution without bubbles.

### Electrical measurements

**Figure 5:**
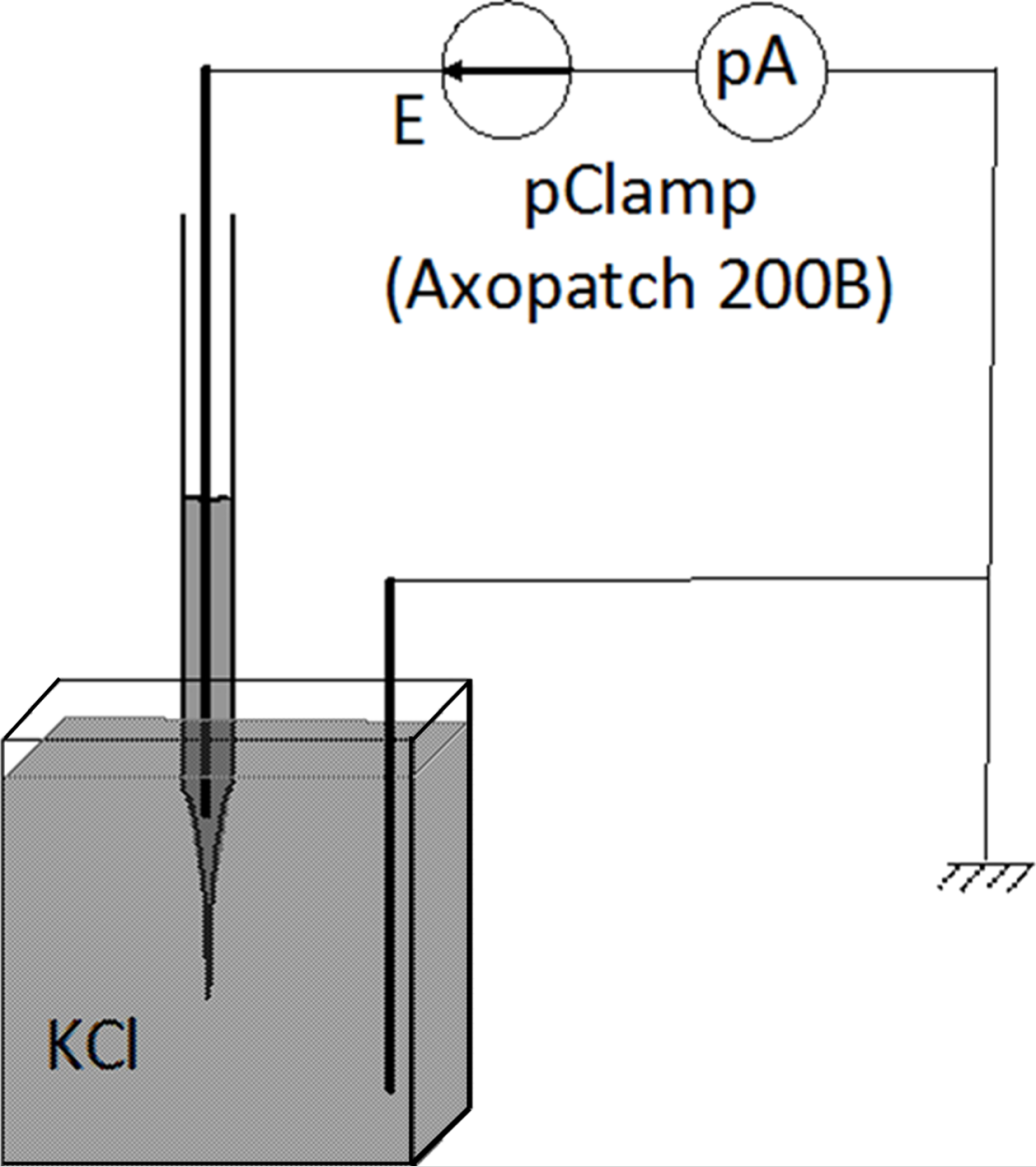
Electrical measurement: one Ag/AgCl electrode is introduced into the large part of the capillary loaded with KCl electrolyte and connected to the head-stage of the Axopatch 200B, the tip of the capillary is plunged into the KCl bath of same concentration, a second Ag/AgCl electrode is plunged into this bath and directly connected to ground. The entire electrical measurement setup is placed under a Faraday cage.

To ensure that electrolyte concentration reaches its nominal value throughout the nanopipette, electromigration is performed in an electrical measurement setup described in Figure 5. When the electrolyte is in the large part of the capillary, the nanopipette is plunged into a tank full of the same electrolyte. One Ag/AgCl electrode is inserted into the capillary and another into the tank. The nanopipette electrode is connected to the pre-amplifier of a pClamp (Axopatch 200B) and the entire electrical measurement setup is placed under a Faraday cage to prevent electrical noise.

## Additional Information

The dynamic microdistillation technique was patented in 2013: B. Tinland, E. Guirleo (Salançon), patent WO 2013079874 A1, “Method and device for filling nanopipettes via dynamic microdistillation”.

## References

[1] Wei C, Bard A J, Felberg S W (1997). Anal Chem.

[2] Hennig S, van de Linde S, Lummer M, Simonis M, Huser T, Sauer M (2015). Nano Lett.

[3] Steinbock L J, Otto O, Chimerel C, Gornall J, Keyser U F (2010). Nano Lett.

[4] Singh K P, Kumar M (2011). J Appl Phys.

[5] Ivanov A P, Actis P, Jönsson P, Klenerman D, Korchev Y, Edel J B (2015). ACS Nano.

[6] Clarke R W, Piper J D, Ying L, Klenerman D (2007). Phys Rev Lett.

[7] Ren R, Zhang Y, Nadappuram B P, Akpinar B, Klenerman D, Ivanov A P, Edel J B, Korchev Y (2017). Nat Commun.

[8] Zweifel L P, Shorubalko I, Lim R Y H (2016). ACS Nano.

[9] Sze J Y Y, Kumar S, Ivanov A P, Oh S-H, Edel J B (2015). Analyst.

[10] Perry D, Momotenko D, Lazenby R A, Kang M, Unwin P R (2016). Anal Chem.

[11] Momotenko D, Cortés-Salazar F, Josserand J, Liu S, Shao Y, Giraud H H (2011). Phys Chem Chem Phys.

[12] Sa N, Baker L A (2013). J Electrochem Soc.

[13] Umehara S, Pourmand N, Webb C D, Davis R W, Yasuda K, Karhanek M (2006). Nano Lett.

[14] Salançon E, Tinland B (2013). J Colloid Interface Sci.

[15] Simonis M, Hennig S (2017). Imaging Microsc.

[16] Ying L, Bruckbauer A, Zhou D, Gorelik J, Shevchuk A, Lab M, Korchev Y, Klenerman D (2005). Phys Chem Chem Phys.

